# Loss of heterochromatin and retrotransposon silencing as determinants in oocyte aging

**DOI:** 10.1111/acel.13568

**Published:** 2022-02-15

**Authors:** Peera Wasserzug‐Pash, Rachel Rothman, Eli Reich, Lital Zecharyahu, Oshrat Schonberger, Yifat Weiss, Naama Srebnik, Yaara Cohen‐Hadad, Amir Weintraub, Ido Ben‐Ami, Hananel Holzer, Michael Klutstein

**Affiliations:** ^1^ Institute of Dental Sciences Faculty of Dental Medicine The Hebrew University of Jerusalem Jerusalem Israel; ^2^ IVF Unit Department of Obstetrics and Gynecology Shaare Zedek Medical Center and Faculty of Medicine Hebrew University of Jerusalem Jerusalem Israel; ^3^ Department of Obstetrics and Gynecology Hadassah‐Hebrew University Medical Center Kiryat Hadassah Jerusalem Israel

**Keywords:** epigenetic aging, heterochromatin, meiosis, oocytes, reproductive aging, retrotransposons

## Abstract

Mammalian oocyte quality reduces with age. We show that prior to the occurrence of significant aneuploidy (9M in mouse), heterochromatin histone marks are lost, and oocyte maturation is impaired. This loss occurs in both constitutive and facultative heterochromatin marks but not in euchromatic active marks. We show that heterochromatin loss with age also occurs in human prophase I‐arrested oocytes. Moreover, heterochromatin loss is accompanied in mouse oocytes by an increase in RNA processing and associated with an elevation in L1 and IAP retrotransposon expression and in DNA damage and DNA repair proteins nuclear localization. Artificial inhibition of the heterochromatin machinery in young oocytes causes an elevation in retrotransposon expression and oocyte maturation defects. Inhibiting retrotransposon reverse‐transcriptase through azidothymidine (AZT) treatment in older oocytes partially rescues their maturation defects and activity of the DNA repair machinery. Moreover, activating the heterochromatin machinery via treatment with the SIRT1 activating molecule SRT‐1720, or overexpression of Sirt1 or Ezh2 via plasmid electroporation into older oocytes causes an upregulation in constitutive heterochromatin, downregulation of retrotransposon expression, and elevated maturation rates. Collectively, our work demonstrates a significant process in oocyte aging, characterized by the loss of heterochromatin‐associated chromatin marks and activation of specific retrotransposons, which cause DNA damage and impair oocyte maturation.

## Graphical abstract

Our work has shown that (a) with age, oocytes’ chromatin becomes less, and less heterochromatic. Silencing and compacting modifications (Presented as colorful hexagons) are gradually lost, changing transcriptional regulation and chromatin structure. (b) The loss of silencing and compaction of heterochromatin results in the loss of transcriptional regulation, when genome accessibility enabling the upregulation of genes that are regularly silent (in blue) and retrotransposons (in red). (c) Retrotransposon activity causes retrotransposition and DNA damage. (d) We suggest that the early stages of reproductive aging are the outcome of the gradual loss of repression. The harmful activity of activated retrotransposons eventually leads to the loss of oocyte functionality and a reduction in fertility.

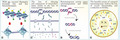

## 1 INTRODUCTION

Reproductive aging is defined as the age‐related loss of fertility due to increasing damage to the reproductive and other systems. Oocytes themselves accumulate damage in an age‐related manner (Igarashi et al., 2015; Miao et al., 2009) and deteriorate to the point where they are non‐functional. In human, females this occurs at a relatively early age, before the onset of aging in other organs and tissues. In our era of increased rate of delayed childbearing, it is becoming crucial to understand the mechanisms underlying the compromised quality of oocytes with age.

Aging is systemic multifactorial process that is hard to define by a single time point or aspect. Reproductive aging, likewise, happens gradually and affects multiple aspects of oocyte function. A well‐studied example of such an aspect is the rise with age of aneuploid oocytes (Hassold & Hunt, 2001; Nagaoka et al., 2012). One of the causes for this is due to precocious sister chromatid separation and resulting non‐disjunction (Gruhn et al., 2019). This occurs because of a decrease in sister chromatid cohesion due to the loss and inability to re‐load Cohesin complexes containing Rec8 on chromosomes (Burkhardt et al., 2016; Gruhn et al., 2019; Hodges et al., 2005; Lister et al., 2010; Revenkova et al., 2010). Despite the study of this important mechanism, it is clear that additional factors contribute to oocyte aging. The decrease in number of mature oocytes retrieved from ovaries occurs before the onset of aneuploidy and is one example of these additional factors. Overall, female fertility itself is in decline much before chromosome non‐disjunction is observed in oocytes (Merriman et al., 2012). For deeper understanding of this aging process, it will be of importance to identify more factors that contribute to this aging process, and to map the effect of the different factors with time.

Changes in epigenetic regulation of gene expression and chromosome structure have been recognized as contributors to aging, and epigenetic changes during aging have been listed among the "hallmarks of aging" (Lopez‐Otin et al., 2013). The loss of heterochromatin histone marks has been associated with the aging process in many systems and tissues (Djeghloul et al., 2016; Jeon et al., 2018; Keenan et al., 2020; Zhang et al., 2015). It was shown that epigenetic changes occur in mouse oocytes of advanced maternal age (Manosalva & Gonzalez, 2010; Marshall et al., 2018; Vazquez et al., 2020; Yue et al., 2012), at ages where aneuploidy is considerable. However, the mechanisms that are altered by these changes, and the ways they affect the different aspects of oocyte aging are yet to be explored. The consequences of heterochromatin de‐regulation in aging may be related to the activated transcription of transposable elements (TE) in the genome, and their subsequent effect on genome stability and cellular integrity. This was shown to occur in several organisms and systems (Chen et al., 2016; De Cecco et al., 2013; Dennis et al., 2012; Patterson et al., 2015; Tarallo et al., 2012). Currently, it is unclear whether TE are activated in older oocytes, and whether, and when exactly, TE expression is involved in oocyte aging and epigenetics.

In this work, we study the role of heterochromatin loss in the aging of oocytes. We show that heterochromatin loss in oocytes can be detected at an age of 9 months in mice, when low aneuploidy rates are present, but a decrease in oocyte quality is evident, as previously reported. (Merriman et al., 2012, Figure 1). We show that these changes are characterized by the loss of repressive histone marks, elevation of specific retrotransposon mRNA transcription, elevated processing of repeated sequences and retrotransposons, and increased activation of the DNA repair machinery. Treatment of oocytes with chemical compounds that inhibit heterochromatin formation can mimic the effect of aging and cause a decrease in oocyte maturation rates and elevation in L1 retrotransposon activity and DNA damage. Importantly, we find that the effect of heterochromatin loss and L1 retrotransposon activity on oocyte maturation with age is partially reversible through treatment of oocytes with AZT, a SIRT1 activating molecule—SRT‐1720, or overexpression of Sirt1 or Ezh2 in older oocytes. Treatment with AZT does not prevent epigenetic failure in older oocytes while the other interventions do. This fact demonstrates that the epigenetic effect is upstream to retrotransposon activation at this stage of the aging process.

**FIGURE 1:**
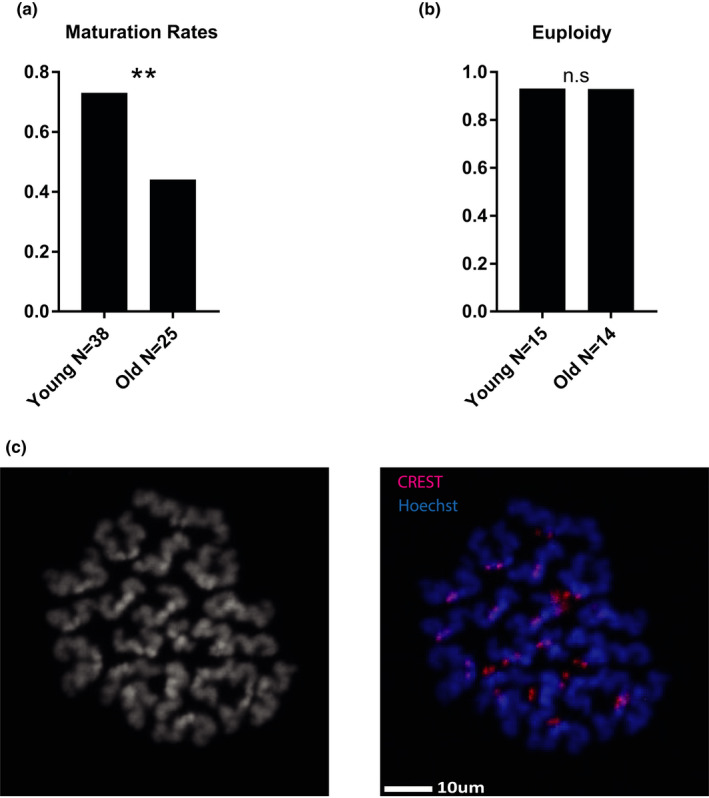
9‐month‐old oocytes show maturation defects but are not uneuploid: (a) In vitro maturation outcomes of naïve young (2‐month‐old) and old (9‐month‐old) oocytes, collected at Prophase I arrest and released simultaneously to meiosis. Oocytes were fixed and stained for DNA to assess entry into MII (Z test for 2 proportions *p* = 0.00135, animals used for this experiment *N*_young_ = 3, *N*_old_ = 3), see Methods Appendix S1 (b) Young (2‐month‐old) and old (9‐month‐old) oocytes chromosomes were spread at prophase of MII. Sister chromatid pairs were counted for each cell to assess ploidy. (Z test *p* = 0.554, animal used for experiment *N*_young_ = 3, *N*_old_ = 4) (c) Representative image of metaphase II chromosome spread, with and without CREST staining

## 2 RESULTS

**FIGURE 2:**
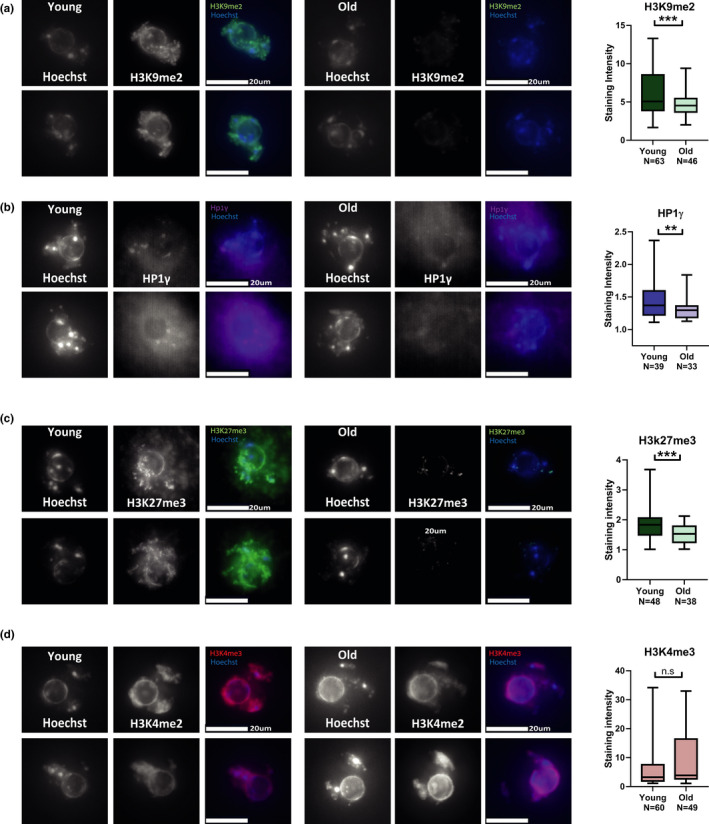
9‐month‐old oocytes lose heterochromatin marks: (a–d) In situ staining images and staining intensity analysis for chromatin marks and proteins in oocytes from young (2‐month‐old) and old (9‐month‐old) females. Oocytes were stained and imaged during prophase I arrest by in situ immunofluorescence (see Methods). Intensity of staining was quantified for each image, and statistical analysis was performed on the numerical values (see Methods). Constitutive heterochromatin marks and proteins were measured by staining for (a) H3K9me2 (*t* test *p* < 0.0001, animals used for experiment *N*_young_ = 4, *N*_old_ = 4) and (b) HP1γ (*t* test *p* = 0.0146, animals used for experiment *N*_young_ = 3, *N*_old_ = 3). (c) To examine facultative heterochromatin oocyte were stained and imaged for H3K27me3(*t* test *p* = 0.0005, animals used for experiment *N*_young_ = 3, *N*_old_ = 5) (d) Euchromatin staining was performed by staining for the marker H3K4me3 (*t* test *p* = 0.657, animals used for experiment *N*_young_ = 3, *N*_old_ = 6) (e–f)

In order to investigate the status of heterochromatin histone marks in oocytes at an age before the onset of aneuploidy, we compared the levels of specific epigenetic markers by immunofluorescence (IF) in prophase I‐arrested oocytes of unstimulated (i.e., not super‐ovulated) 2‐month‐old (young) and 9‐month‐old (old) mouse females. According to previous studies (Koehler et al., 2006; Manosalva & Gonzalez, 2010; Merriman et al., 2012), mouse oocyte aneuploidy at the age of 9 months is low and not significantly different than in young mice. However, other fertility associated traits such as the level of oocyte maturation at 9 months by IVM (in vitro maturation), number of oocytes found in the oviduct after superovulation and fecundity are already markedly reduced. The mouse strain we use (RCC‐C57BL/6JHsd, see methods) in the present study shows consistent findings with this decline in maturation rates that were 27% lower in old compared with young mice, and aneuploidy rates, which were identical between both age groups (Figure 1a–c). We then proceeded to immunostaining of the oocytes for constitutive heterochromatin markers H3K9me2 and HP1γ. Both markers' levels decrease significantly with age when assessed by in situ immunofluorescent staining (Figure 2a,b, see methods for further details). In addition, the level of the facultative heterochromatin mark H3K27me3 also reduces with age when assessed by in situ immunofluorescent staining (Figure 2c).

Prophase I oocytes in mice and human have two main chromatin configuration forms, which characterize two developmental stages. The first chromatin configuration in maturing oocytes, is termed non‐surrounded nucleolus (NSN), and is characterized by a diffuse chromatin configuration. Gradually, with oocyte growth, chromatin is organized around the nucleolus in a ring‐shaped structure, a configuration termed surrounded nucleolus (SN). The transition from NSN to SN is accompanied by multiple cellular changes, including comprehensive histone modification changes, and transcription silencing that continues throughout oocyte maturation and early embryogenesis (Wasserzug‐Pash & Klutstein, 2019; Bouniol‐Baly et al., 1999). Interestingly, the defect in heterochromatin modification with age is much more pronounced as the oocyte proceeds to the SN stage—when the oocyte undergoes transcriptional shutdown (Figure S1). However, facultative heterochromatin decrease occurs uniformly in all prophase I arrest stage oocytes (both NSN and SN), showing that some age‐related epigenetic defects can already be seen at an early oocyte maturation stage (Figure S1). Importantly, transcription is still active in Prophase I‐arrested oocytes as was shown by BrU incorporation into RNA (Bouniol‐Baly et al., 1999), with complete transcriptional shutdown occurring only later during the oocyte maturation process. This was verified by plasmid electroporation experiments showing expression of eGFP in electroporated Prophase I‐arrested oocytes (Figure S2; electroporation of nucleic acids into oocytes was already performed by others (Hirata et al., 2019; Grabarek et al., 2002).

To investigate whether the decrease in repressive marks occurs as a result of nucleosome or histone loss and to control for differences in oocyte staining capacity between young and old oocytes, we stained oocytes by in situ immunofluorescence for the active chromatin marks H3K27Ac and H3K4me3. Importantly, no significant difference was detected between young and old oocytes for these two chromatin marks (Figure 2d, Figure S3). These results show that the loss of histone marks is specific to heterochromatin modifications and that no detectable histone loss has occurred in old oocytes.

**FIGURE 3:**
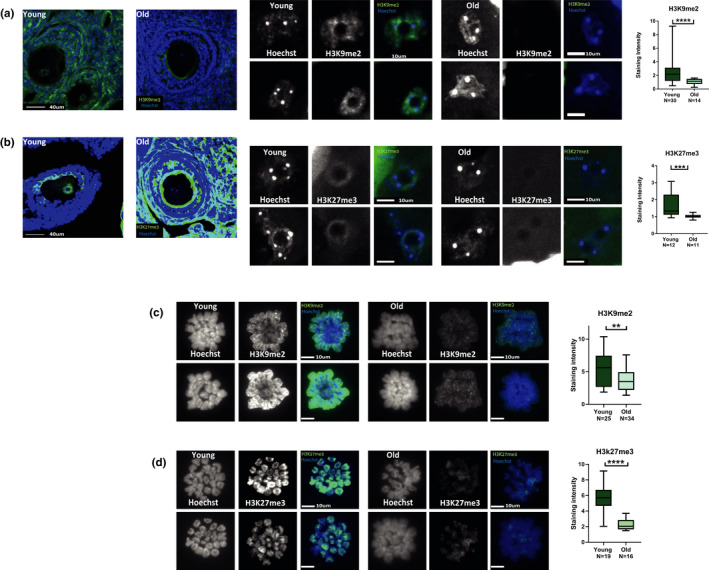
Heterochromatin loss appear uniformly in diverse oocytes populations: IHF staining was performed on ovaries cryosections from young (2‐month‐old) and old (9‐month‐old) female mice. Loss of heterochromatin was detected in the intact oocytes in both (a) H3K9me2 (*t* test < 0.0001, animals used for experiment *N*_young_ = 3, *N*_old_ = 3) and (b) H3K27me3 staining. (MW = 0.0008, animals used for experiment *N*_young_ = 3, *N*_old_ = 3). Staining of MI metaphase chromosomes and staining intensity analysis from young (2‐month‐old) and old (9‐month‐old) females (see Methods) was performed for the following markers: (c) H3K9me2 (*t* test *p* = 0.008, animals used for experiment *N*_young_ = 3, *N*_old_ = 5) and (D)H3K27me3 (MW *p* < 0.0001, animals used for experiment *N*_young_ = 3, *N*_old_ = 4)

To achieve information about wider range of oocytes population and to avoid possible confounding effects from immunofluorescence staining procedures, we performed IHF on mouse ovaries cryosections. Using this method, we measured heterochromatin levels in a wider variety of oocyte stages and sizes than those achieved by manual collection of oocytes. Heterochromatin loss with age was also apparent in ovary cryosections as demonstrated by the lower level of H3K9me2 and H3K27me3 (Figure 3a,b) in the aged ovaries oocytes compared with the young group. This trend was observed in all the oocytes at all sizes. Moreover, after characterizing epigenetic changes in aging non‐mature oocytes, we also explored if the epigenetic defect persists till the later stages of oocyte maturation. To do this, we investigated heterochromatin markers of oocytes at metaphase of MI by performing chromosome spreads followed by immunostaining. Spreads were stained for H3K9me2 and for H3K27me3 (Figure 3c,d). In these experiments, a significant drop in signal was observed in older oocytes. This result shows that the epigenetic defects that occur in older oocytes persist till the meiotic divisions. Since the spread experiments enable the visualization of the entire chromosome, we also asked whether the decrease in repressive chromatin marks occurs on specific loci in the genome, or whether the decrease is uniform along the entire chromosome. No significant pattern of specific preference was found in our analysis. Despite the limited ability to discern specific regions of the genome by microscopy, this result suggests that the decrease in heterochromatin is widespread and includes many regions in the genome (Figure S4).

**FIGURE 4:**
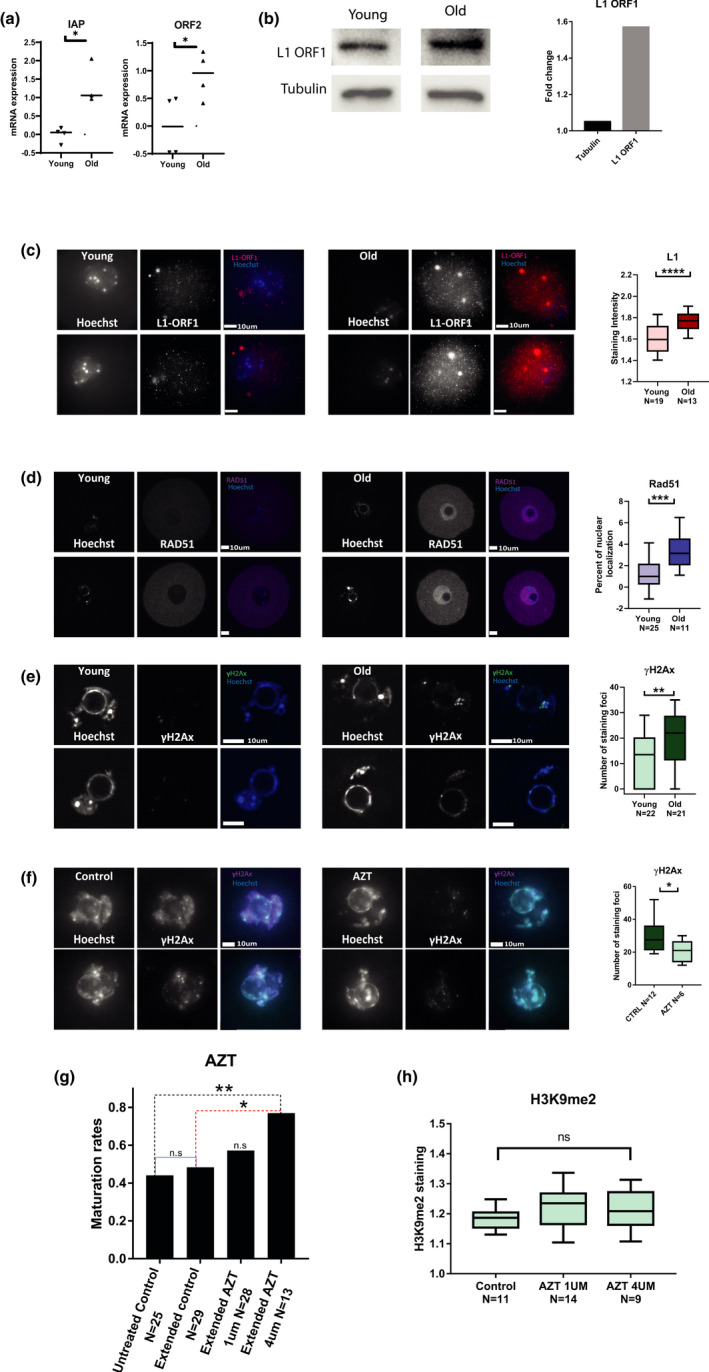
Old oocytes show elevated activity of retrotransposons L1 and IAP and increased DNA damage: (a) Transcriptional retrotransposon activity was measured using qRT‐PCR for L1 retrotransposon (MW *p* = 0.01, animals used for experiment *N*_young_ = 10, *N*_old_ = 10) and IAP elements mRNA (MW *p* = 0.03, animals used for experiment *N*_young_ = 10, *N*_old_ = 10) (see Methods) in young (2‐month‐old) and old (9‐month‐old) prophase I‐arrested mouse oocytes. (b) Upregulation of L1 retrotransposon protein was measured by Western blotting for L1‐ORF1p in from young (2‐month‐old) and old (9‐month‐old) prophase I‐arrested mouse oocytes. (*N*_young_ = 6, *N*_old_ = 8) (c) Upregulation of L1 retrotransposon activity was measured by in situ staining for L1‐ORF1p in from young (2‐month‐old) and old (9‐month‐old) prophase I‐arrested mouse oocytes. (Shapiro Wilk *p* < 0.05, t test *p* < 0.0001, *N*_young_ = 3, *N*_old_ = 3). (d) Accumulation of DNA damage with age was assessed by in situ staining for Rad51 and quantification of its nuclear localization (see Methods) in young (2‐month‐old) and old (9–month‐old) prophase I‐arrested mouse oocytes (MW *p* < 0.0005, *N*_young_ = 3, *N*_old_ = 5). (e) Another measure for DNA damage was performed by in situ staining for γH2AX (MW *p* = 0.0085, *N*_young_ = 3, *N*_old_ = 5) that was quantified by the number of nuclear foci in old and young prophase I‐arrested mouse oocytes (see Methods). (f) To investigate the phenotype of aged oocytes after retroviral activity inhibition, oocytes were treated with AZT and then stained for γH2A and the number of nuclear foci in AZT treated and untreated control old prophase I‐arrested oocytes was quantified (MW *p* < 0.0274 animals used for experiment *N* = 3) (g) The maturation efficiency of old oocytes treated with two different doses of AZT and matured in vitro was measured by proportion of oocytes which successfully completed MII after treatment (*Z* test *p* = 0.0102) (see Methods). Because incubation with the drug required longer incubation in IBMX than normal, an extended incubation without AZT was also added as control (h) Quantification of in situ staining intensity for H3K9me2 in control old oocytes and old oocytes treated with two different doses of AZT (see Methods)

Histone post‐translational modifications are a key component in transcriptional regulation, and specifically in the silencing of transposable elements (TE) that compose ~50% of mammalian genomes. The activation of TE may pose a significant threat to the genome of the cell, and the regulation and silencing of the activity of TE is becoming recognized as a hallmark of cellular integrity in health and disease (Bravo et al., 2020; Enriquez‐Gasca et al., 2020; Kohlrausch et al., 2021). To investigate the consequences of the loss of heterochromatin on the transcription of TE in old oocytes, we examined RNA expression from two retrotransposon families: long interspersed nuclear element‐1 (L1) and intracisternal A particle (IAP), which were shown to be expressed in the germline (Crichton et al., 2014; Dupressoir & Heidmann, 1996; Trelogan & Martin, 1995). Oocytes from older females had a roughly 2‐fold increased expression of both L1 and IAP transcripts compared with young oocytes as assessed by quantitative RT‐PCR (Figure 4a). An elevation in retrotransposon‐derived protein synthesis in aged oocytes was detected by Western blot for a protein of the L1 retrotransposon, L1‐encoded ORF1p (Figure 4b). We confirmed these findings by immunostaining oocytes. L1‐ORF1p was detectable at low levels in young oocytes and increased in intensity in older oocytes (Figure 4c). In accordance with increased activity of these TE, older oocytes show increased recruitment of DNA repair machinery indicative of DNA damage. This was shown by elevated Rad51 nuclear localization in older oocytes and higher presence of γH2Ax foci (Gasior et al., 2006; Figure 4d,e). Foci of γH2Ax are considered a bona fide marker of DNA damage and repair (Fernandez‐Capetillo et al., 2004). An elevated nuclear localization of Rad51 is also a marker for DNA damage and was observed in HeLa and HCT116 cells after treatment with ionizing radiation (Gildemeister et al., 2009).

We asked whether an inhibition of reverse transcription activity of retrotransposons, and as a result, inhibition of retrotransposition in older oocytes improves their cellular integrity and maturation ability. To do that, we matured in vitro older oocytes with or without the addition of azidothymidine (AZT), a reverse‐transcriptase inhibitor, that was shown to inhibit retrotransposon activation in fetal oocytes (Malki et al., 2014; Tharp et al., 2020). Being a thymidine kinase 1 inhibitor, AZT also presented a toxic effect on cell growth in some cell lines (Lynx et al., 2008). However, previous studies on oocytes did not report a toxic effect in this system. Nevertheless, we used lower concentrations than those reported to be toxic (see Methods). Our results (Figure 4g) show that the relatively low level of maturation of older oocytes in vitro can be partially rescued and show an elevation in maturation in up to 28.6% more mature oocytes after incubation in 4 µM AZT. We also show that DNA damage is also reduced by AZT treatment as presented by the reduction in γH2Ax loci in these oocytes (Figure 4f). Despite these effects, the AZT treatment did not alter heterochromatin levels in the older oocytes (Figure 4h), hinting that heterochromatin loss may be upstream to retrotransposons elevation.

**FIGURE 5:**
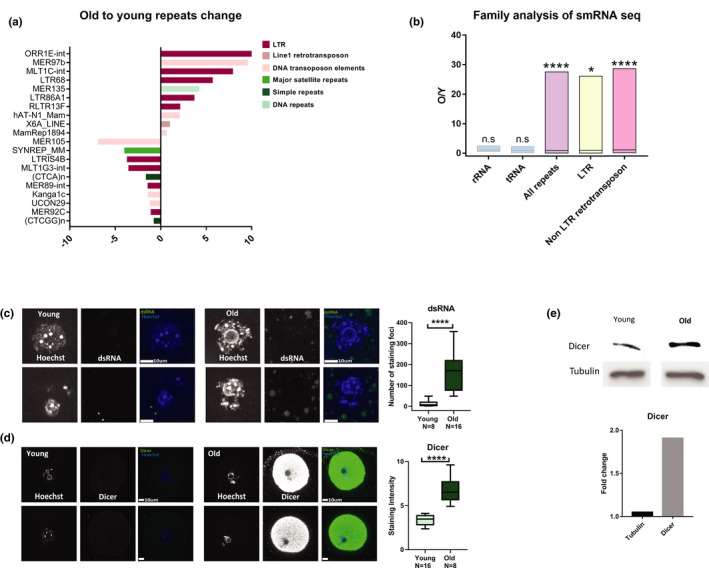
Old oocytes show elevated retrotransposon RNA processing activity: Small RNA‐seq was performed in order to quantify the differential presence of small RNAs in young (2‐month‐old) and old (9‐month‐old) oocytes (see Methods and Table S1). (a) Identification of 10 repeat types that are most upregulated or downregulated in old oocytes. Note that the predominant types of repeats that were overexpressed are retrotransposon RNA molecules, while the list of under‐expressed repeat types also includes other types of repeats. (b) Family based analysis of smRNA. The ratio in sequencing reads between old and young in the transcripts of each repeat family are plotted. tRNA and rRNA are used as controls. (c, d) To quantify global RNA processing activity in the aged oocytes, we stained for dsRNA (MW *p* < 0.0001, *N*_young_ = 3, *N*_old_ = 5) (c) and Dicer (MW *p* < 0.0001, *N*_young_ = 3, *N*_old_ = 5) (d) in young and old prophase I‐arrested mouse oocytes. (e) Dicer elevation was confirmed using Western blotting (*N*_young_ = 6, *N*_old_ = 8)

In addition to the expression of retrotransposons in older oocyte, we investigated whether we also see evidence for increased processing of retrotransposon RNA. An elevation in retrotransposon expression can result in elevated retrotransposon mRNA expression and its translation into protein but also in its elevated processing. RNA processing systems such as RNAi regulate gene expression by posttranscriptional targeting of mRNA coming from regulated regions. The processing system inhibits unregulated transcription activity by Dicer‐mediated digestion of transcripts into small RNAs and activation of various downstream targeting and silencing complexes such as Argonaute proteins (Cross et al., 2019; Hogg et al., 2011; Kanakamani et al., 2021). Increased activity of the RNA processing machinery indicates the occurrence of a loss of transcription regulation, and it is possible to identify the specific loci where this loss has occurred by the analysis of the small RNA repertoire. We therefore performed small RNA sequencing on oocytes from young and old females. We sequenced (in duplicate) RNA molecules from 17 to 167 bp (median of 20–80 percentiles, see Methods and Figure S5) with a median molecule size of 18 bp for the young oocytes and 106 for the older oocytes (Figure S5). A measurement of the sizes of the small RNA fragments was done computationally (see Methods). A prominent peak of ribosomal RNA at 155 bp can be observed in both samples. The difference in small RNA sizes between young and old oocytes shows an elevated presence of RNA fragments of sizes that are not typical of Dicer products and could originate from spurious transcription due to the loss of genomic repression of heterochromatin. Small RNAs can originate from different regions in the genome. However, the difference in RNA expression between the young and old oocytes was evident specifically in the group of small RNAs coming from genomic repeats (usually marked by heterochromatin). Most repeat types remained unchanged between young and older oocytes (83%). 4.6% of repeat types were overexpressed and 12.1% were under‐expressed more than 2‐fold in older oocytes (Table S1). However, when looking at which repeat types were changed‐ the list of overexpressed repeat types in old oocytes (Figure 5a) shows that most RNA molecules are derived from retrotransposons, while in the under‐expressed repeats additional repeat types are present. When we compared the fold change of expression in our data between different families of RNA molecules, we saw that while in tRNAs and rRNAs there is no elevation in older oocytes over young oocytes, in genomic repeats in general, and specifically in retrotransposons, there was a significant elevation in transcripts in older oocytes vs. young oocytes (Figure 5b). In agreement with more retrotransposon mRNA and more retrotransposon‐derived small RNA from some of the repeats in older oocytes, we also see a significant elevation in the signal of dsRNA molecules in older oocytes (Figure 5c). Moreover, the Dicer protein, an enzyme which participates in the processing of retrotransposons (Soifer et al., 2005; Svobodova et al., 2016) showed an increase as well, both by immunostaining and Western blot analysis (Figure 5d,e). We therefore conclude that the loss of epigenetic silencing in old oocytes results in accompanied by transcriptional activity and processing of retrotransposons in the genome.

**FIGURE 6:**
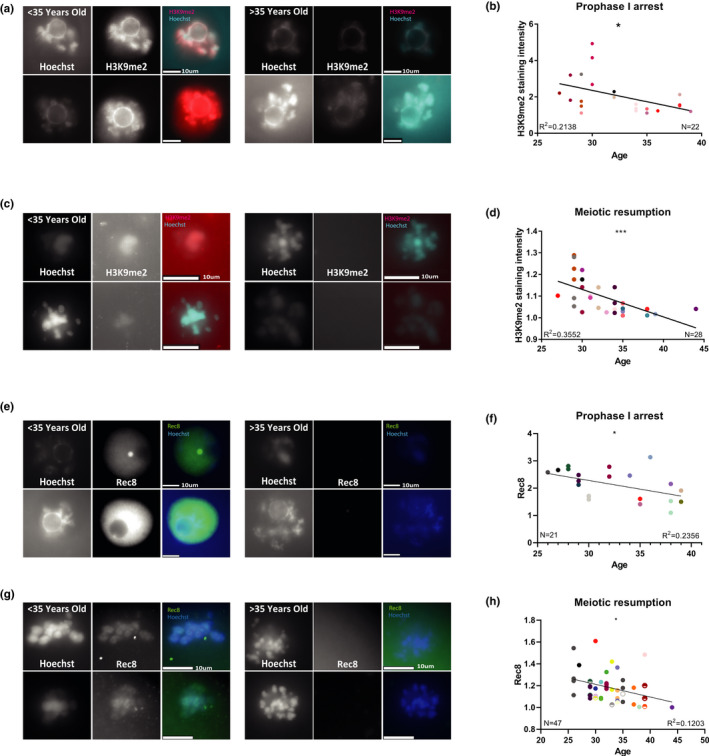
Human oocytes show a reduction of the H3K9me2 heterochromatin marker, and REC8 meiotic cohesion marker with age: To study the changes in heterochromatin with age in Human oocytes, human GV‐arrested oocytes were stained by immunofluorescence for H3K9me2 (see text and Methods). Oocytes were retrieved from 33 women, with average of 2.2 oocyte per woman. Analysis was performed separately for oocytes that were stained during prophase I arrest or arrested later during meiosis. (a) Examples of stained (for H3K9me2) prophase I‐arrested oocytes from patients under 35‐year‐old and over 35‐year‐old (b) Oocytes arrested during meiosis also show a reduction of signal intensity with age with a linear correlation, (*N* = 28, *F* test *p* = 0.0008). The oocytes of each patient are in a different color (for a given age, several patients with more than one oocyte may be represented). (c) Examples of stained (for H3K9me2) prophase I oocytes after meiotic resumption, from patients under 35‐year‐old and over 35‐year‐old. (d) Prophase I oocytes show a reduction of signal intensity with age with a linear correlation. (*N* = 21, *F* test *p* = 0.03) (e) Examples of in situ staining of prophase I‐arrested human oocytes (*N* = 21) for Rec8 meiotic cohesion subunit. (f) These oocytes show a linear reduction of signal intensity with age (*F* test *p* = 0.02). (g) Examples of in situ staining of human prophase I oocytes after meiotic resumption (*N* = 47) for Rec8. (h) These oocytes show a linear reduction of signal intensity with age (*F* test *p* = 0.01)

Since some significant differences in epigenetic regulation exist between mice and human (Hanna et al., 2018), we wanted to investigate whether heterochromatin loss with maternal age also occurs in human oocytes. For this purpose, we investigated the heterochromatin of oocytes from IVF treatments. Oocytes were retrieved from 33 patients, with an average of 2.2 oocytes per patient. As a general rule, prophase I‐arrested oocytes are not used by clinics for fertilization, but to make sure the oocytes we used for research could not mature in vitro, we waited another 24 h with the oocytes in medium, before we fixed the immature oocytes and stained them. Therefore, these oocytes likely represent a subset of human oocytes which are meiotically incompetent (for details of ethics approval, see Methods). Indeed, fixed oocytes that were stained for DNA visualization were arrested in several developmental stages (Figure 6). Further, we stained oocytes by in situ immunofluorescent staining for H3K9me2. Stringent QC criteria were applied to the staining results in order to exclude oocytes with fragmented or damaged genomes (see Methods). We found that in oocytes arrested both at the prophase I‐arrest stage and after release from meiotic arrest, there was an age‐dependent decrease in H3K9me2 signal fitting a linear decreasing curve (Figure 6a–d). As control, we also stained human oocytes for the Rec8 meiotic cohesin (specificity of the antibody is shown in Figure S6). This staining also shows (Figure 6e–h) a decreasing, age‐dependent trend, as has been shown before for mouse and human cohesin (Chiang et al., 2010; Liu & Keefe, 2008; Tsutsumi et al., 2014).

**FIGURE 7:**
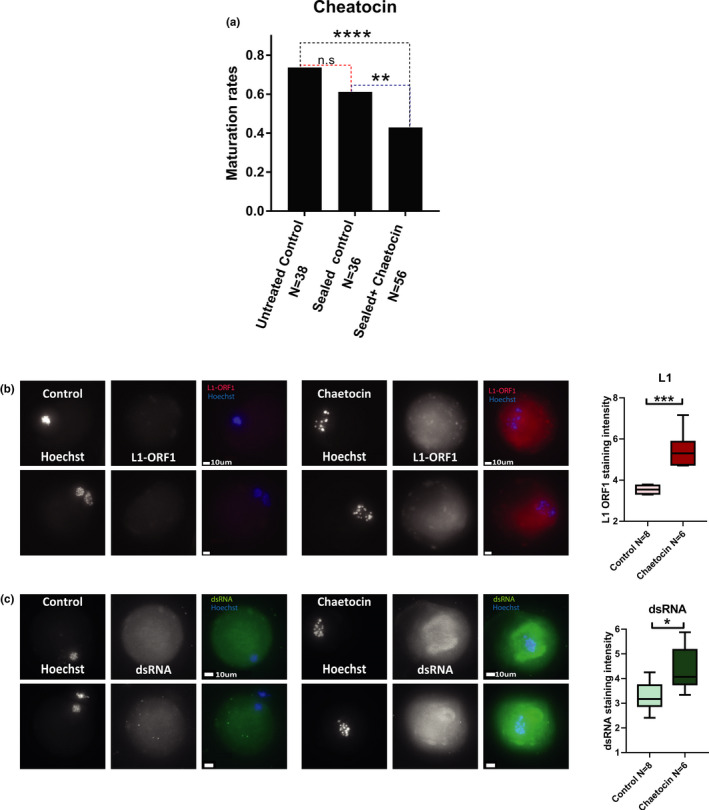
Treatment of oocytes with Chaetocin mimics natural aging: Young prophase I‐arrested mouse oocytes (9 animals were used for this experiment) were released to resume meiosis in a medium supplemented with Chaetocin that inhibits heterochromatin formation (see Methods). (a) The resulting phenotype was measured by the assessment of maturation efficiency of treated oocytes compared with untreated controls (*Z* test *p* = 0.008). Incubation with Chaetocin requires sealing of the plate for bio‐safety reasons. Therefore, an additional control with a sealed plate but without the drug was added (see Methods). (b) Activation of L1 retrotransposons following Chaetocin treatment was measured by immunofluorescence for L1‐ORF1p in young MII mouse oocytes with or without Chaetocin (MW *p* = 0.0007) (c) To measure RNA processing activity in Chaetocin‐treated oocytes, in situ staining was performed for dsRNA in young MII mouse oocytes with or without Chaetocin. (MW *p* = 0.02)

In order to show a causal link between heterochromatin deterioration and maturation defects of old oocytes, we treated young mouse oocytes with chemicals known to affect epigenetic regulation. The Suvar39h1/2 enzyme is essential for heterochromatin formation, and Chaetocin has been characterized as a specific and effective Suvar39h1/2 inhibitor (Greiner et al., 2005). We therefore treated 2‐month‐old oocytes with Chaetocin (Greiner et al., 2005) at 0.5 µM in vitro for 18 h (Bertoldo et al., 2020). Staining for H3K9me2 decreased after the treatment by staining of chromosome spreads at metaphase of MI (Figure S7). However, in situ staining of MI‐treated oocytes for H3K4me3 presented no significant change, indicating that heterochromatin manipulation with Chaetocin does not interfere with other chromatin regulation aspects (Figure S7). The assessment of the maturation efficiency of the oocytes after treatment with Chaeotocin shows reduction of 18% in the oocytes that properly mature after treatment (Figure 7a). Staining of treated oocytes by in situ immunofluorescence was performed after treatment. L1‐encoded ORF1p shows an increase in staining intensity (Figure 7b). Consistently, dsRNA staining presented a significant increase in the treated compared with the untreated control oocytes (Figure 7c).

Since heterochromatin that is characterized by H3K9me2 and binding of HP1γ is also associated with a histone de‐acetylation on H3K27 (Naruse et al., 2020; Wang et al., 2019), we sought to investigate whether inhibiting the de‐acetylation of histones in oocytes will cause a similar effect. Previous reports also showed that treatment with a histone deacetylase (HDAC) inhibitor affects oocyte maturation in vitro (Jin et al., 2014). We thus treated young oocytes with the HDAC inhibitor Trichostatin A (TSA, 100 nm for 4 h of arrest and then for 18 h until MII) (Yoshida et al., 2003). In vitro treated oocytes show an increase in H3K27Ac and also a decrease in H3K9me2 by in situ staining linking different heterochromatin pathways such as H3K27 de‐acetylation and H3K9 methylation in oocytes (Figure S8). TSA‐treated young oocytes show similar maturation defects to Chaetocin‐treated young oocytes and unchanged staining for H3K4me3 (Figure S8). Interestingly, TSA‐treated oocytes do not show a L1‐encoded ORF1p elevation in overall signal level. Instead, L1‐ORF1p accumulates in nuclei of treated oocytes, perhaps showing that de‐acetylation activity is central to specific stages in retrotransposon maturation processes (Figure S8), causing enhanced nuclear recruitment of the L1 protein when de‐acetylation processes fail to occur. However, DNA damage response is elevated in TSA‐treated oocytes as shown by the elevated Rad51 nuclear localization in treated oocytes (Figure S8). Collectively, these results show that epigenetic manipulation, in H3K9 methylation or H3K27 acetylation pathways causes the inability of young oocytes to mature, and mimics natural aging.

**FIGURE 8:**
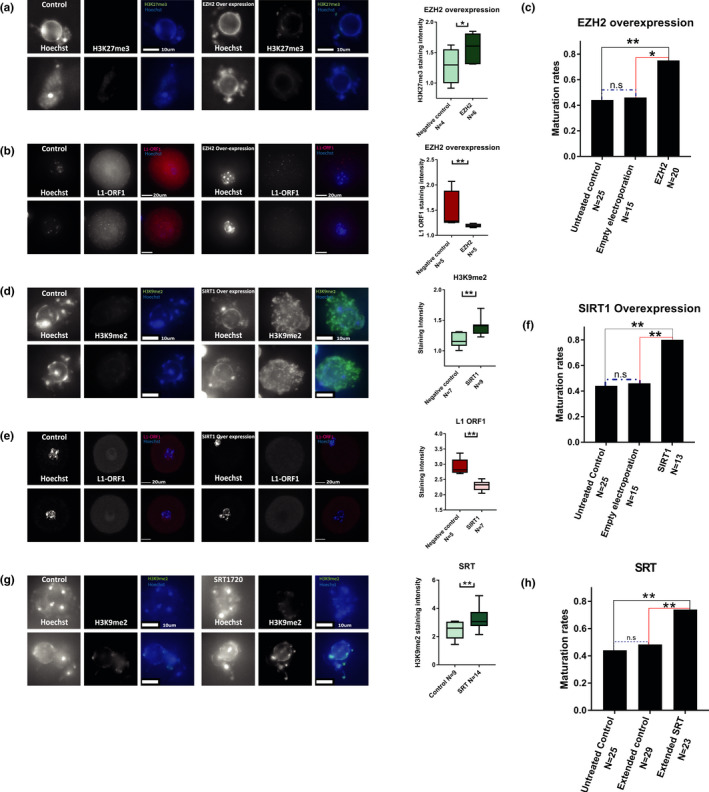
Aged oocytes recovery occurs when heterochromatin is enhanced: Overexpression of chromatin modifier enzymes was performed in old (9‐month‐old) prophase I‐arrested oocytes. (a) Heterochromatin recovery in EZH2 electroporated oocytes was measured by H3K27me3 staining (MW *p* = 0.03, 4 animals used for experiment) (b) Reduction in retrotransposon activity in treated oocytes was observed by staining for L1‐‐ORF1 (MW *p* = 0.03, 3 animals used for experiment). (c) Cellular functionality was measured by successful MII accomplishment in EZH2 electrporated oocytes compared with empty electroplation control. (*Z* test *p* = 0.019, 7 animals used for experiment) (d) Heterochromatin recovery in SIRT1 electroporated oocytes was measured by H3K9me2 staining (MW *p* = 0.0026 3 animals used for experiment). (e) Reduction in retrotransposon activity in treated oocytes was observed by staining for L1‐ORF1 (MW *p* = 0.0013 5 animals used for experiment). (f) Cellular functionality was measured by successful MII accomplishment in SIRT1 electrporated oocytes compared with empty electrporation control. (*Z* test *p* = 0.0086, 7 animals used for experiment) (g) Quantification of staining intensity for H3K9me2 in control old oocytes and old oocytes treated with SRT‐1720 (MW *p* = 0.0079) (h) Maturation efficiency of old oocytes treated with SRT‐1720 and matured in vitro was measured by proportion of oocytes which succeed to complete MI after treatment. (*Z* test *p* = 0.0078, animals used for experiment *N* = 6). As above, since incubation with the drug required longer IVM incubation than normal, an extended incubation without SRT‐1720 was also added as control

To further strengthen the causal link between heterochromatin loss, retroviral activity, and oocytes maturation defects in older oocytes, we tested whether the elevation of heterochromatin by gene overexpression could reverse oocyte aging phenotypes. To do that, we used plasmid electroporation as above (Figure S2), to overexpress EZH2, an enzymatic subunit of the PRC2 complex that catalyzes H3K27 methylation (Margueron & Reinberg, 2011), and SIRT1, an NAD+ dependent HDAC (Michishita et al., 2005; Vaquero et al., 2007) (see Methods). Treated oocytes presented a significant increase in heterochromatin. As expected, oocytes electroporated with EZH2 showed an elevation in H3K27 methylation staining compared with an empty electroporation control (Figure 8a), and oocytes electroporated with SIRT1 plasmid presented an increase in H3K9 methylation. (Figure 8d). In both groups, the increase in heterochromatin led to significant decrease in L1‐ORF1 staining (Figure 8b,e), showing that when heterochromatin is recovered in these oocytes they re‐gain their ability to silence retroviral activity. Finally, treated oocytes presented increased maturation rates (Figure 8c,f) compared with empty electroporation controls. To confirm these results with another manipulation targeting Sirtuins, we cultured aged oocytes with the SIRT1 activating molecule SRT‐1720. Treated oocytes had a similar phenotype to the overexpression experiment above. SRT‐1720 treatment triggered heterochromatin elevation, that resulted in recovered maturation ability of treated cells (Figure 8g,h). Notably, similar results were previously shown by supplementation of older oocytes with the SIRT1 associated metabolic cofactor nicotinamide adenine dinucleotide (NAD+), which caused rejuvenated oocyte quality in aged animals (Bertoldo et al., 2020). Taken together, these results show that heterochromatin loss occurs upstream of retrotransposon activity, and that heterochromatin loss may be reversible in older oocytes.

## 3 DISCUSSION

We show a reduction in repressive chromatin marks with age in mammalian oocytes. This trend can be observed in both mouse and human oocytes. In mouse oocytes, we show that heterochromatin loss occurs at a significant rate even before the onset of aneuploidy. In a series of experiments, we demonstrate here that the reduction in repressive marks is associated with oocyte maturation defects, expression of L1 retrotransposons, an increase in dsRNA and Dicer presence in the cytoplasm and an elevation in recruitment of DNA repair proteins. Using the compounds Chaetocin and TSA that target epigenetic modifications, we show that an artificial reduction in repressive marks in young oocytes also causes expression of L1 retrotransposons, an elevation in DNA damage and maturation defects. Inhibition of retrotransposons reverse‐transcriptase activity through AZT treatment partially rescues oocytes maturation defects but does not impact their heterochromatin loss. However, heterochromatin can be recovered by overexpression of chromatin modifiers SIRT1 and EZH2. Using this approach, retrotransposon activity is re‐inhibited and the maturation ability is recovered. Similar results were obtained using the SIRT1 activating molecule SRT‐1720. This shows that the age‐related heterochromatin defects act upstream of retrotransposon activation. Our working model (Figure S9) is therefore that due to external damage (from exposure to stressors, such as toxins and radiation) and erosion with time, heterochromatin modified histones are lost from chromatin and replaced by non‐modified nucleosomes. This process may be enhanced by the inability of the oocyte to replenish its heterochromatin due to the elongated cell cycle arrest past the S phase, when heterochromatin re‐enforcement normally occurs (He et al., 2017; Jahn et al., 2018). Another possible contributor to heterochromatin loss may be changes of histone loading and unloading dynamics. These possible contributions should be addressed in future experiments. Heterochromatin loss with time leads to a cascade of molecular events that eventually lead to a transcriptional de‐regulation. Silencing of wide genomic domains is eventually lost, and L1 retrotransposon RNA is transcribed. This in turn causes an elevation in DNA damage, and a decrease in oocyte maturation ability. This elevation in L1 retrotransposon activity with age in oocytes could have survived in evolution in order to serve as a selection mechanism to eliminate non‐functional oocytes from the oocyte pool. A similar mechanism has been previously suggested to occur during fetal oocyte attrition (Malki et al., 2014; Tharp et al., 2020). We thus hypothesize that heterochromatin loss serves as an oocyte associated quality assurance mechanism—only allowing the maturation of oocytes that have not sustained high levels of DNA damage.

## 4 MATERIALS AND METHODS

### 4.1 Animals

RCC‐C57BL/6JHsd female mice were used for the experiment. For the young group, we used 7‐11‐week‐old mice, and for the old group we used 8–10 month‐old mice. The experiment was approved by the institutional ethics committee, approval number: MD‐19‐15938‐3. For more details, see Appendix S1.

### 4.2 Mouse Oocyte In vitro maturation

After Euthanasia, ovaries were collected and dissected in L‐15 medium (011151A) and supplemented with 200 μm IBMX (I7018) to prevent meiotic progression. Prophase I‐arrested oocytes were identified by visualization of the typical germinal vesicle, and collected under a binocular using a Stripper (MD‐MXL3‐STR‐CGR). After collection, oocytes were transferred into α‐MEM medium (22561021) supplemented with IBMX covered with mineral oil (M8410‐1l) to prevent evaporation, for recovery time of 25 min at 37° in a 5% CO2 incubator, and then washed in IBMX free α‐MEM medium to initiate meiosis. The oocytes were incubated in α‐MEM under oil in the incubator for 6 h for prophase I or incubated for 19 h for metaphase II. To assess maturation rates, oocytes were incubated for 19 h after release and then fixed and stained by Hoechst to examine the entry into the second meiotic division. See below amendments for drug treatments.

### 4.3 Mouse oocytes collection for in situ immunofluorescence

After Euthanasia, ovaries were collected and dissected in M2 medium (M7167). Oocytes were collected from the pool of 3 animals at least (numbers of each experiment are noted in legends). Prophase I‐arrested oocytes were collected under a binocular using a Stripper and washed in hyaluronidase (H4272‐30MG) to remove granulosa cells and acidic Tyrode's Solution (T1788) to remove the Zona Pellucida. The oocytes were then fixed and stained. For more details, see Appendix S1.

### 4.4 Chromosome spreads and immunofluorescence

Prophase I‐arrested oocytes were collected from ovaries as above and incubated for 6 h to reach the first prophase or for 17 h to reach second meiosis prophase as described above. Matured oocytes were washed in M2 medium and then in acidic Tyrode's Solution to remove the Zona Pellucida. 15 min before the desired time for chromosome spread, oocytes were kept in hypotonic solution, composed of FBS (F7524) diluted in DDW in 1:1 ratio. Oocytes were then spread in spreading solution (1% PFA buffered to 9.2 pH, supplemented with 0.15% Triton X‐100 and 0.03% DDT) on Superfrost plus slides (32090003). To assess ploidy, prophase II spreads were stained with Hoechst to visualize chromatin and CREST to visualize the centromeres, and the imaged chromosome were manually counted.

### 4.5 Mouse ovaries IHF

Ovaries were collected, washed in PBS, and fixed with shaking at 4° for 24 h in 4% PFA. After fixation, the ovaries were kept in 30% sucrose with shaking at 4° overnight. The tissue was kept in Tissue‐Tek OCT Compound (4583) at −80°. Cryosectioning was performed on a Leica CM 1950 Cryostat, and sections were applied on superfrost plus slides. Sections were cultured in PBS containing 1%FBS and 0.1% Triton X‐100 for blocking and stained. For more details, see Appendix S1.

### 4.6 Imaging and quantification

In order to analyze the images quantitatively, a projection of maximum intensity was created. To measure staining intensity, every assessed region was normalized to an equal‐size region in the background (outside the area of interest). The intensity score was generated by dividing the intensity in the area of interest by that of the background. For more details, see Appendix S1.

### 4.7 Small RNA sequencing

~50 oocytes (number was matched between groups in every experiment) from young and old females (each in triplicate) were collected from ovaries as above and dissected in medium M2, and after the granulosa cells and the Zona Pellucida were removed (as mentioned above), the oocytes were inserted into 1 ml of TRIzol (15596026). RNA extraction was done according to the TRIzol reagent user guide, with extraction in 10 µl of nuclease‐free water.

Amplification was performed by the SMARTer^®^ smRNA‐Seq Kit for Illumina^®^‐12 Rxns, TAKARA 635029. Size selection was performed using Agencourt AMPure XP Beads, and sequencing was performed on a NextSeq machine. For more details, see Appendix S1.

### 4.8 Differential expression

Normalization and differential expression analysis were done with the DESeq2 package. Results are detailed in Table S1. Results were corrected for batch effect and further normalized between samples to a control gene expression (Rian (Hatada et al., 2001)). Raw data submitted to GEO under accession GSE159789. Sequencing results appear in Table S1. For more details, see Appendix S1.

### 4.9 qRT‐PCR

After Euthanasia, mouse ovaries were collected as above. 40–60 (number was matched between groups in every experiment) prophase I‐arrested oocytes were collected and washed in hyaluronidase (H4272‐30MG) to remove granulosa cells, and then washed extensively in M2 until all the granulosa cells were removed from the oocytes. Oocytes were transferred to TRIzol (15596026) and chloroform solution for RNA precipitation. Reverse transcription was performed using iScript™ Reverse Transcription Supermix for RT‐qPCR (1708891) according to the manufacturer's recommendations. iTaq™ Universal SYBR^®^ Green Supermix (1725124) was used for amplification reactions. Quantitative PCR measurements were taken using a CFX96 C1000 BioRad machine.

### 4.10 Mouse oocytes western blot

After Euthanasia, ovaries were collected and dissected in L‐15 medium (011151A) supplemented with 200 μm IBMX (I7018) to prevent meiotic progression. 101 prophase I‐arrested oocytes were collected from each and washed in hyaluronidase (H4272‐30MG) to remove granulosa cells, and then washed extensively in L‐15 until all the granulosa cells were removed from the oocytes. The clean oocytes were then washed in PBS until all the culture media was gone, and transferred into RIPA buffer (1%NP‐40, 0.1%SDS, 50 mM Tris, 150 mM NaCl, 0.5% sodium deoxycholate, 1 mM EDTA, 1X cOmplete™ Protease Inhibitor Cocktail 11836145001), and kept on ice. Protein extraction was performed by boiling the lysate in Laemmli buffer (1610747) 335 mM 2‐mercptoethanol. The lysates were fractionated by 10% acrylamide SDS gel under reducing conditions and transferred to a nitrocellulose membrane (Millipore) using a transfer apparatus according to the manufacturer's protocol (Bio‐Rad). Blots were developed with an ECL system according to the manufacturer's protocol (Bio‐Rad). Results were collected using ChemiDoc XRS+System from Bio‐Rad.

### 4.11 Oocyte Electroporation

Ovaries were collected and dissected in L‐15 medium supplemented with 200 μm IBMX to prevent meiotic progression as described above. Prophase I oocytes were washed with acidic Tyrode's Solution, and transferred into EC‐002 electroporation cuvettes containing 100 μl of clean L‐15 medium for the control group and 50–200 ng/ul, lonza pmaxGFP (DMC00054), or the gene expressing plasmids for the experimental group. Electroporation was done in Nepagene NEPA21 electroporator. The electroporated oocytes were washed briefly and then cultured in IBMX supplemented α‐MEM. 24 h after electroporation oocytes were fixed and stained with reported antibodies. CMVp‐SIRT1 plasmid was reported by the Reinberg group (Vaquero et al., 2007), and the CMVp‐EZH2 plasmid was reported by the Sartorelli group (Caretti et al., 2004).

### 4.12 Human oocytes

Human prophase I‐arrested oocytes that were retrieved during IVF treatment were incubated for 24 h before determination that they remained at this state and did not mature to become fully grown MII oocytes. After informed consent was signed (following IRB approval 0020‐16‐SZMC) oocytes were treated by piercing of the Zona Pellucida by the embryologists in order to increase permeability. The oocytes were then fixed in 4% PFA the day after retrieval, for immunofluorescence was performed as described above. For more details, see Appendix S1.

## CONFLICT OF INTEREST

The authors declare that they have no conflict of interest.

## AUTHOR CONTRIBUTIONS

MK and PWP conceived the project. PWP, RR, and ER performed mouse oocytes experiments. OS, YW, NS, YCH, AW, IBA, and HH contributed to human oocyte experiments and analysis. LZ assisted with data analysis. PWP analyzed all data and built the figures. PWP and MK wrote the manuscript. MK supervised the study and obtained the funding.

## Supporting information

Fig S1

Fig S2

Fig S3

Fig S4

Fig S5

Fig S6

Fig S7

Fig S8

Fig S9

Table S1

Appendix S1

## Data Availability

All reported original microscopy data are available upon request. Raw sRNA‐seq has been deposited to GEO under accession GSE159789. Sequencing analysis results appear in Table S1.
