# Supplementary material for: Loss of heterochromatin and retrotransposon silencing as determinants in oocyte aging
Source: Aging Cell. 2022 Feb 15;21(3):e13568. doi: 10.1111/acel.13568 (PMC8920445; doi:10.1111/acel.13568)
Supplement: Supplementary file 11 — Appendix S1 [file ACEL-21-e13568-s001.docx]

**Detailed experimental procedures:**

Animals: RCC-C57BL/6JHsd female mice were used for the experiment. For the young group we used 7-11 weeks old mice, and for the old group we used 8-10 months old mice. Mice were used after an acclimation period of at least 3 days after shipment. The experiment was approved by the institutional ethics committee, approval number: MD-19-15938-3. All mice were housed at the Hebrew University AAALAC-accredited and NIH-accredited SPF facility.

Mouse Oocyte In-vitro maturation: After Euthanasia, ovaries were collected and dissected in L-15 medium (011151A) and supplemented with 200um IBMX (I7018) to prevent meiotic progression. Prophase I arrested oocytes were identified by visualization of the typical germinal vesicle, and collected under a binocular using a Stripper (MD-MXL3-STR-CGR). After collection, oocytes were transferred into α-MEM medium (22561021) supplemented with IBMX covered with Mineral oil (M8410-1l) to prevent evaporation, for recovery time of 25 min at 37° in a 5% CO2 incubator, and then washed in IBMX free α-MEM medium to initiate meiosis. The oocytes were incubated in α-MEM under oil in the incubator for 6 hours for prophase I or incubated for 19 hours for metaphase II. To assess maturation rates, oocytes were incubated for 19 hours after release and then fixed and stained by Hoechst to examine the entry into the second meiotic division. See below amendments for drug treatments.

Mouse oocytes collection for in-situ immunofluorescence: After Euthanasia, ovaries were collected and dissected in M2 medium (M7167). Oocytes were collected from pool of 3 animals at least (numbers of each experiment are noted in legend). Prophase I arrested oocytes were collected under a binocular using a Stripper and washed in hyaluronidase (H4272-30MG) to remove granulosa cells and acidic Tyrode's Solution (T1788) to remove the Zona Pellucida. The oocytes were fixed using PFA 4% (15710) for 20 min, and then quenched in PBS supplemented with 10mM glycine and 1% BSA.

Chromosome spreads and immunofluorescence: Prophase I arrested oocytes were collected from ovaries as above and incubated for 6 hours to reach the first prophase or for 17 hours to reach second meiosis prophase as described above. Matured oocytes were washed in M2 medium and then in acidic Tyrode's Solution to remove the Zona Pellucida. 15 min before the desired time for chromosome spread, oocytes were kept in hypotonic solution, composed of FBS (F7524) diluted in DDW in 1:1 ratio. Oocytes were then spread in spreading solution (1% PFA buffered to 9.2 pH, supplemented with 0.15% Triton X-100 and 0.03% DDT) on Superfrost plus slides (32090003). To assess ploidy, prophase II spreads were stained with Hoechst to visualize chromatin and CREST to visualize the centromeres, and the imaged chromosome were manually counted.

In situ immunofluorescence: Permeabilization was performed using 0.1% Triton X-100. Cells were cultured in PBS contains 5% BSA for blocking, and first and secondary antibodies were diluted in 0.1 Tween 20 (P9416)/PBS containing 5% BSA. The immune-stained cells were mounted in Vectashield (H-1000) mounting medium containing 80 nM of Hoechst (33342), and sealed on a slide using an Imaging spacer (GBL654002).

Mouse ovaries IHF: Ovaries were collected, washed in PBS and fixed with shaking at 4° for 24 hours in 4% PFA. After fixation the ovaries were kept in 30% sucrose with shaking at 4° overnight. The tissue was kept in Tissue-Tek OCT Compound (4583) in -80°. Cryo-sectioning was performed on a Leica CM 1950 Cryostat, and sections were applied on superfrost plus slides. In order to capture most of the oocytes' chromatin, that spans over ~30um in the Z plane per cell, every section was taken in 20um thickness. To avoid having the same oocytes (50-70 um~) in two different sections on the same slide, every slide contained sections that are at least 80µm apart. Before staining, the slides were dried overnight at room temperature, and washed extensively in PBS to remove OCT. Sections were cultured in PBS containing 1%FBS and 0.1% Triton X-100 for blocking. All antibodies were diluted in a concentration of 1:100 in blocking solution. Slides were mounted in Vectashield mounting medium containing 80 nM of Hoechst, and sealed with nail polish.

Antibodies: For heterochromatin staining we used antibodies for H3K9me2 (Abcam, ab1220, working dilution 1:400), H3K27me3 (Abcam, ab205728, 1:100) HP1γ (Abcam, ab227478, 1:50). For euchromatin markers we used antibodies for H3K27ac (Cell signaling, 8173s, 1:100) and H3K4me3 (Cell signaling, 11960s, 1:50). To track retroviral activity and transcription regulation loss we used antibodies for L1-ORF1p (Abcam, ab216324, 1:100), dsRNA (SCICONS,10020200, 1:500) and Dicer (Abcam, ab167444, 1:200). Meiotic Cohesin was stained with an antibody against REC8 (Abcam, ab192241 1:200), and DNA damage was assessed using antibodies against RAD51(Sigma, PC130, 1:200), and γH2Ax (Sigma, 0563625UG, 1:500). GFP expression was measured using anti-GFP antibody (Rockland 600-101-215, 1:250). Centromere visualization was achieved using CREST antibody (INC 15-235-0001, 1:100).
For western blot primary antibodies include; GAPDH (Abcam, ab8245 1:3000), REC8 (Abcam, ab192241, 1:4000), L1-ORF1p (Abcam, ab216324, 1:1000), Dicer (Abcam, ab167444, 1:1000), and Tubulin (T5168 1:4000) Anti-rabbit and anti-mouse horseradish peroxidase-conjugated secondary antibodies were used (Goat Anti-Rabbit IgG H&L (HRP) ab6721 (1:10,000), and Rabbit Anti-Mouse IgG H&L (HRP) ab6728(1:10,000) or Goat Anti-Mouse IgG H&L (HRP) ab97040(1:10,000).

Imaging and quantification: Oocytes were imaged using the Ti-Eclipse Nikon system, with an Andor Zyla nsc05537 camera. Confocal imaging was used when light imaging failed to obtain a clear image, using Yokogawa W1 Spinning Disk on Motorized fluorescent microscope Ti2E, with 2 SCMOS ZYLA cameras or Zeiss LSM710. Every cell was imaged at multiple planes. In order to analyze the images in a quantitatively, a projection of maximum intensity was created. To measure staining intensity, every assessed region was normalized to an equal-size region in the background (outside the area of interest). The intensity score was generated by dividing the intensity in the area of interest by that of the background.

Small RNA sequencing:
RNA extraction and amplification: ~50 oocytes (number was matched between groups in every experiment) from young and old females (each in triplicate) were collected from ovaries as above and dissected in medium M2, and after the granulosa cells and the Zona Pellucida were removed (as mentioned above), the oocytes were inserted into 1 ml of TRIzol (15596026). RNA extraction was done according to the TRIzol reagent user guide, with extraction in 10 µl of nuclease-free water.
Amplification was performed by the SMARTer® smRNA-Seq Kit for Illumina®-12 Rxns, TAKARA 635029. Size selection was performed using Agencourt AMPure XP Beads, by adding 50ul of sample to 100ul of beads solution and eluting the DNA bound to the beads (selecting transcripts below 200 bp). Sequencing was performed at the Ein-Kerem campus interdepartmental equipment park on a NextSeq machine.

Trimming and filtering of raw reads: The NextSeq base-calls files were converted to fastq files using the bcl2fastq program with default parameters (without trimming or filtering applied at this stage). Raw reads (fastq files) were inspected for quality issues with FastQC. Following that, and according to the SMARTer smRNA-Seq library construction protocol, the first 3 bases of R1 reads were discarded (positions for template switching), the reads were quality-trimmed at both ends, poly-G sequences (NextSeq’s no signal), adapter sequences, and poly-A sequences were removed from the 3' end, and finally low quality reads were filtered out. Cutadapt was used for trimming sequences from the end of reads, with parameters that included using a minimal overlap of 1, allowing for read wildcards, and filtering out reads that became shorter than 15 nt. Final filtering by quality was performed using the fastq_quality_filter program of the FASTX package, with a quality threshold of 20 at 90 percent or more of the read's positions.

Alignment and counting: The processed reads were aligned to the mouse transcriptome and genome with TopHat. The genome version was GRCm38, with annotations from Ensembl release 99. Quantification was done with htseq-count.

Differential expression: Normalization and differential expression analysis were done with the DESeq2 package. Results are detailed in Table S1. Results were corrected for batch effect and further normalized between samples to a control gene expression (Rian (Hatada et al., 2001)). Raw data submitted to GEO under accession GSE159789. Sequencing results appear in supplementary table 1.

qRT-PCR: After Euthanasia, mouse ovaries were collected and dissected in M2 medium (M7167) with 200um IBMX (I7018) to prevent meiotic progression as above. 40 to 60 (number was matched between groups in every experiment) prophase I arrested oocytes were collected and washed in hyaluronidase (H4272-30MG) to remove granulosa cells, and then washed extensively in M2 until all the granulosa cells were removed from the oocytes. Oocytes were transferred to TRIzol (15596026) and Chloroform solution for RNA precipitation, treated with RNAse free DNAse (E0013-1D3) to remove DNA and then RNA was purified by using Ampure beads (A63881). Reverse transcription was performed using iScript™ Reverse Transcription Supermix for RT-qPCR (1708891) according to the manufacturer’s recommendations. iTaq™ Universal SYBR® Green Supermix (1725124) was used for amplification reactions. Quantitive PCR measurements were taken using a CFX96 C1000 BioRad machine.

Mouse oocytes western blot: After Euthanasia, ovaries were collected and dissected in L-15 medium (011151A) supplemented with 200um IBMX (I7018) to prevent meiotic progression. 101 prophase I arrested oocytes were collected from each and washed in hyaluronidase (H4272-30MG) to remove granulosa cells, and then washed extensively in L15 until all the granulosa cells were removed from the oocytes. The clean oocytes were then washed in PBS until all the culture media was gone, and transferred into RIPA buffer (1%NP-40, 0.1%SDS, 50mM Tris, 150mM NaCl, 0.5% Sodium Deoxycholate, 1mM EDTA, 1X cOmplete™ Protease Inhibitor Cocktail 11836145001), and kept on ice. Protein extraction was performed by boiling the lysate in Laemmli buffer (1610747) 335mM 2-mercptoethanol. The lysates were fractionated by 10% acrylamide SDS gel under reducing conditions and transferred to a nitrocellulose membrane (Millipore) using a transfer apparatus according to the manufacturer’s protocol (Bio-Rad). Blots were developed with an ECL system according to the manufacturer’s protocol (Bio-Rad). Results were collected using ChemiDoc XRS+ System from Bio-Rad.

Drug treatments: Ovaries were collected and dissected in L-15 medium supplemented with 200um IBMX to prevent meiotic progression as described above. Prophase I arrested oocytes were collected using a Stripper as described above. After collection, oocytes were transferred to α-MEM medium supplemented with IBMX + the desired drug covered with Mineral oil to prevent evaporation, for 4 hours at 37°in a 5% CO2 incubator (Since Chaetocin and IBMX have a cross reactivity with each other, the pause was not performed in Chaetocin experiments). After the pause, oocytes were washed in IBMX-free α-MEM that contains the drug to initiate meiosis. 19 hours after oocytes were released from IBMX, oocytes were fixed in PFA, permeabilized, and sealed as described above. Chaetocin is a hazarzous substance. For biosafety reasons, Chaetocin supplemented plates and matched controls were placed in a sealed chamber during incubation time. Drugs in use were: Trichostatin A (TSA) T1952, Chaetocin (sc-200893), SRT1720 (biovision 2772) and Zidovudine (AZT) (PHR1292-1G).

Oocyte Electroporation: Ovaries were collected and dissected in L-15 medium supplemented with 200um IBMX to prevent meiotic progression as described above. Prophse I oocytes were washed with acidic Tyrode's Solution, and transferred into EC-002 electroporation cuvettes containing 100ul of clean L-15 medium for the control group and 50-200ng/ul, lonza pmaxGFP (DMC00054) or the gene expressing plasmids for the experimental group. Electroporation was done in Nepagene NEPA21 electroporator. The electroporated oocytes were washed briefly and then cultured in IBMX supplemented α-MEM. 24 hours after electroporation oocytes were fixed and stained with reported antibodies. CMVp-SIRT1 plasmid was reported by the Reinberg group (Vaquero et al., 2007), and the CMVp-EZH2 plasmid was reported by the Sartorelli group (Caretti, Di Padova, Micales, Lyons, & Sartorelli, 2004).

Human in vitro fertilization (IVF) protocols: Patients were treated with one of two protocols for ovarian stimulation determined by their physician. Long agonist protocol started in the luteal phase of the menstrual cycle, or antagonist protocol started on the second day of the menstrual cycle. For the controlled ovarian hyperstimulation in both protocols either recombinant FSH preparation, recombinant FSH and LH or HMG (human menopausal gonadotrophin) were used. Dosage was determined individually for each patient according to BMI and ovarian reserve parameters. Ovarian response was monitored by serial ultrasound examinations and the evaluation of serum E2 levels, then gonadotropin doses adjustment was done as required. Human chorionic gonadotropin (HCG) was administered when at least 3 leading follicles were 17-18 mm in diameter. Oocyte retrieval was performed 36 hours following HCG administration using transvaginal ultrasound guided approach.

Human oocytes: Human prophase I arrested oocytes that were retrieved during IVF treatment were incubated for 24h before determination that they remained at this state and did not mature to become fully grown MII oocytes. After informed consent was signed (following IRB approval 0020-16-SZMC) oocytes were treated by piercing of the Zona Pellucida by the embryologists in order to increase permeability. The oocytes were then fixed in 4% PFA the day after retrieval, for 20min at room temperature and then quenched in PBS supplemented with 10mM glycine and 1% BSA. Immunofluorescence was performed as described above. Analysis was performed separately for prophase I arrested oocytes, recognized by the typical germinal vesicle and dispersed chromatin configuration, and for oocytes which arrested after meiotic resumption, recognized by lack of germinal vesicle and chromatin condensation. Oocytes that entered the second meiotic division, recognized by chromatin division and polar body extrusion were excluded from the data.

REC8 antibody specificity: HCT116 human colon carcinoma cell line cells were maintained in Dulbecco's Modified Eagle's medium (DMEM, Sigma), supplemented with 10% fetal bovine serum (FBS), 1% PenStrep (100 U/mL Penicillin and 100 μg/mL Streptomycin) in a 37 °C incubator (5% CO2). The cell lines were authenticated at the Biomedical Core Facility of the Technion, Haifa, Israel. REC8 tagged with Enhanced Green Fluorescent Protein (EGFP), and EGFP only (vector pEGFP-N1, plasmid no. 170528HM8137-6 from BD Biosciences) were used. The QIAprep® Spin miniprep kit by Qiagen (cat-27104, Germany) was used to extract the plasmid. All cells were transfected 24h after initial plating. Transfections were performed using the PolyJet™ In Vitro DNA Transfection Reagent (cat-SL100688, USA) with a 3:1 (transfection reagent: DNA) ratio. 48h after transfection, adherent cells were lysed using hot sample buffer (10% glycerol, 50 mmol/L Tris-HCl pH 6.8, 20% SDS, and 5% 2-mercaptoethanol), and western blot analysis was carried out. The lysates were fractionated by SDS PAGE (Criterion™ TGX (Tris-Glycine eXtended) Stain-Free™ precast gels, Bio-Rad) under reducing conditions and transferred to nitrocellulose membrane (Millipore) using a transfer apparatus according to the manufacturer’s protocol (Bio-Rad). Blots were developed with an ECL system according to the manufacturer’s protocol (Bio-Rad). Results were collected using ChemiDoc XRS+ System from Bio-Rad.

Statistical analysis: Statistical analysis was performed using Excel, R and GraphPad prism. To compare between means- if N>30 or if a Shapiro Wilk test returned insignificant the analysis was performed using a parametric test (t test for single comparison and one way Anova for group comparison). Otherwise, a non-parametric test was used (Mann Whitney (MW) for single comparison and Kruskal Wallis (KW) for group comparison). To compare the difference between groups in transcript presence, ratio-paired T test was used. To compare proportions, Z test for two proportions was used, when normality of parameters was confirmed either by N>30 or by N*P>5 for each of the compared groups. Correlation was calculated using Person's correlation coefficient, and significance of the calculated R was determined using F test.
